# Correlation Between Age, Gender, Tongue Position, Residual Alveolar Jaw Relationship, and Duration of Complete Edentulism in Patients From Benghazi, Libya: A Cross-Sectional Study

**DOI:** 10.7759/cureus.103121

**Published:** 2026-02-06

**Authors:** Fatma M Ali, Raina S Eltawati Salem, Amel M Lefsaay, Sara M Bogazia, Samiyah A Mohammed

**Affiliations:** 1 Diagnosis, Specialized Center for Dental Treatment and Education, Benghazi, LBY; 2 Fixed Prosthodontics, University of Benghazi, Benghazi, LBY; 3 Removable Prosthodontics, University of Benghazi, Benghazi, LBY; 4 Oral Biology, University of Ajdabiya, Ajdabiya, LBY; 5 Dental Public Health and Preventive Dentistry, University of Benghazi, Benghazi, LBY

**Keywords:** complete dentures, complete edentulism, gender differences, residual ridge resorption, tongue position, tooth loss

## Abstract

Purpose

Complete edentulism is a common condition with considerable functional and psychological implications. This study aimed to assess the relationship between gender and various factors, including age, causes of tooth loss, duration of edentulism, tongue position, and residual ridge classification among completely edentulous Libyan patients receiving complete dentures.

Methods

A cross-sectional observational study was conducted on 500 completely edentulous Libyan patients at the Department of Prosthodontics, Faculty of Dentistry, University of Benghazi, Benghazi, Libya, between May 2018 and October 2024. Eligible participants were receiving their first complete dentures for both jaws and had a minimum edentulous period of three months. Data were collected through personal interviews and clinical examinations. Statistical analysis was performed using IBM SPSS Statistics for Windows, Version 20 (Released 2011; IBM Corp., Armonk, NY, USA), with a significance level of p < 0.05.

Results

Of the 500 participants, 65.4% were male and 34.6% female, with a mean age of 68 years. Periodontal disease (49.6%) and dental caries (46.1%) were the most common causes of tooth loss, with a moderate correlation to gender (r = 0.694, p < 0.0001). Males had a longer average edentulous period (11 months) compared to females (4 months), showing a weak correlation (r = 0.432). Class I tongue position was most frequent (73%), as was Class I (orthognathic) ridge relationship (87%). Correlations between gender and tongue position (r = 0.417) and residual ridge classification (r = 0.241) were weak but statistically significant.

Conclusion

The findings highlight a higher prevalence of complete edentulism among older Libyan males, primarily associated with periodontal disease. Tongue position and ridge relationship were generally favorable for denture retention. These results support the need for gender-specific considerations in edentulous patient management.

Clinical relevance

Understanding gender-based differences in edentulism characteristics can enhance treatment planning and prosthetic outcomes in complete denture rehabilitation.

## Introduction

Complete denture rehabilitation is one of the most prevalent and widely accepted prosthodontic treatment options for edentulous patients with systemic, anatomical, or socioeconomic constraints [1]. Successful prognostic outcomes of complete denture therapy are influenced by multiple factors, including patient demographics, age, psychological characteristics, prior denture experience, personal standards and attitudes, residual ridge anatomy, the construction process, denture quality, time-dependent changes, and esthetics [2].

Complete edentulism remains a significant oral public health concern among the elderly and greatly impacts primary care dentistry [3]. Tooth loss results from various factors such as periodontal disease, dental caries, oral cancer, cystic lesions, failed root canal treatments, and trauma. The absence of teeth impairs speech, mastication, and appearance, adversely affecting an individual’s quality of life [4]. Additionally, tooth loss has been shown to differ by sex, with females being more commonly affected [5].

Residual ridge resorption (RRR) is a natural consequence of tooth extraction due to the bone's inherent tendency to resorb. The rate of resorption is highest in the early post-extraction phase, which significantly compromises the prosthetic outcomes for completely edentulous patients [6]. Complete dentures are subjected to varying degrees of displacing forces due to the dynamic environment of the oral cavity, influenced by muscular activity. For optimal function, dentures must remain stable and retained, requiring that the retentive forces exceed those causing displacement [7].

The anatomical and physiological features of the tongue and surrounding oral tissues must be thoroughly evaluated during complete denture planning. An unfavorable resting tongue position can negatively affect the retention and stability of mandibular complete dentures [8]. Numerous factors influence the retention and stability of removable complete dentures, including the condition of the oral mucosa, vestibular sulcus depth, presence of tongue hypertrophy, alveolar bone quantity and quality, bone mineral density, soft tissue resilience, maxillomandibular ridge relationships, neuromuscular coordination, and the physical properties of saliva, such as adhesion, cohesion, and flow [7].

The primary objective of this study was to describe the demographic and clinical distributions of completely edentulous Libyan patients receiving their first complete dentures. The secondary objective was to evaluate associations between gender and clinical/anatomical factors, including tongue position, residual ridge classification, and duration of edentulism.

This study was exploratory in nature, aiming to detect potential associations without pre-specified directional hypotheses, consistent with the study design and statistical analyses performed.

## Materials and methods

A cross-sectional observational study was conducted on completely edentulous Libyan patients who required removable complete dentures to replace missing teeth. This study, the first of its kind in both Benghazi and Libya, was carried out at the Department of Prosthodontics, Faculty of Dentistry, University of Benghazi, Benghazi, Libya.

Prior to commencement, ethical approval was obtained from the Scientific Research Committee at the University of Benghazi (Approval No: 0250). The study was conducted over a period of six years and eight months, from May 5, 2018, to October 12, 2024.

All participants were informed about the study objectives, procedures, and confidentiality measures. Verbal informed consent was obtained from each participant prior to enrollment, in accordance with the ethical approval granted for this observational, non-interventional research. The datasets generated and/or analyzed during the current study are not publicly available due to patient confidentiality and institutional policy. However, de-identified data may be made available from the corresponding author upon reasonable request.

The sample size was calculated as the target population for this study comprised 2,400 completely edentulous Libyan patients who attended the Department of Prosthodontics, Faculty of Dentistry, University of Benghazi, between May 2018 and October 2024. To ensure adequate statistical power and precision, the sample size was calculated using a single population proportion formula: $n_0 = \frac{Z^2 \cdot p \cdot (1 - p)}{e^2}$.

Using this formula, the initial sample size was: $n_0 = \frac{(1.96)^2 \cdot 0.5 \cdot (1 - 0.5)}{(0.05)^2} = 384$.

Since the total population is finite (N = 2,400), a finite population correction (FPC) was applied: $n = \frac{n_0}{1 + \frac{n_0 - 1}{N}} = \frac{348}{1 + \frac{348 - 1}{2400}} \approx 327$.

Thus, the minimum required sample size was 327 participants. To increase precision, account for potential exclusions, and ensure sufficient power for subgroup analyses, 500 participants were ultimately enrolled. This sample size provides adequate power (>80%) to detect moderate associations between gender, age, cause of tooth loss, tongue position, residual ridge classification, and duration of edentulism.

Inclusion criteria consisted of Libyan patients requiring simple removable dentures for both jaws. All participants were receiving complete dentures for the first time and had undergone tooth extractions at least three months prior to enrollment to ensure complete bone healing.

Exclusion criteria included non-Libyan individuals, patients who were not completely edentulous, and medically healthy individuals who were candidates for alternative prosthodontic treatments (such as implant-supported or fixed prostheses) rather than removable complete dentures.

Out of 2,400 individuals initially assessed, 500 cases were selected for inclusion in the study. A total of 1,900 participants were excluded: 826 were medically healthy and received or were eligible for alternative prosthodontic treatments, 676 had insufficient documentation in their clinical history, and 398 were non-Libyan.

Data were collected through face-to-face interviews and clinical examinations. This included demographic information, dental history, and intraoral findings. Each participant provided a personal history and underwent dental and intraoral clinical examinations, which included evaluation of tongue position and residual ridge relationship. These parameters were categorized into three defined sectors. All examinations, including assessment of tongue position and residual ridge classification, were performed by a team of experienced examiners: an assistant professor, associate professors, and a professor registered in the Department of Prosthodontics at the Faculty of Dentistry, University of Benghazi. Examiners were trained and experienced in prosthodontic assessment to ensure consistency and reliability across evaluations.

Tongue position was classified according to Wright's classification. Tongue position in edentulous patients is classified into three types to assess denture stability. Class I: the tongue lies on the floor of the mouth with the tip forward and slightly below the incisal edges of the mandibular anterior teeth. Class II: the tongue is flattened and broadened, but the tip remains in a normal position. Class III: the tongue is retracted and depressed into the floor of the mouth, with the tip curled upward, downward, or assimilated into the body of the tongue based on the resting posture of the tongue relative to the alveolar ridges [8]. Residual ridge classification was recorded as orthognathic, retrognathic, or prognathic [9].

Statistical analysis was performed using IBM SPSS Statistics for Windows, Version 20 (Released 2011; IBM Corp., Armonk, NY, USA). A significance level of p < 0.05 was used, with a 95% confidence interval. The mean and standard deviation were calculated for the numerical variable age, which was also categorized into three age groups. Pearson’s correlation test was used to assess relationships among gender, causes of tooth loss, duration of edentulism, tongue position, and residual ridge relationships. Comparisons between groups were conducted using the Chi-square test.

## Results

These results were derived from the personal and dental histories, as well as clinical examinations, of 500 completely edentulous Libyan patients. Regarding gender distribution, 327 (65.4%) participants were male, while 173 (34.6%) were female. As shown in Table 1, the mean age for both genders was approximately 68 years. A moderate positive correlation was observed between gender and age (r = 0.544). A one-sample Chi-square goodness-of-fit test, assuming an equal distribution, revealed a statistically significant predominance of males among the studied edentulous patients (χ² = 47.43, df = 1, p < 0.001, φ = 0.31). Similarly, the distribution of patients across age groups showed a statistically significant deviation from equality (38-58 years: 124/500, 24.8%; 58-78 years: 212/500, 42.4%; ≥79 years: 164/500, 32.8%; χ² = 23.30, df = 2, p < 0.001, Cramér’s V = 0.22), with the 58-78-year age group representing the largest proportion of cases.

**Table 1:** Personal Demographics of 500 Completely Edentulous Libyan Patients * Statistically significant.

Variable	Category	Observed n (%)	χ²	df	p-value	Effect size
Gender	Male	327 (65.4)	47.43	1	<0.001*	φ = 0.31
	Female	173 (34.6)				
Age group (years)	38-58	124 (24.8)	23.30	2	<0.001*	V = 0.22
	58-78	212 (42.4)				
	≥79	164 (32.8)				

There was no statistically significant association between age group and gender (38-58 years: males 84/327, 25.7%; females 40/173, 23.1%; 58-78 years: males 129/327, 39.4%; females 83/173, 48.0%; ≥79 years: males 114/327, 34.9%; females 50/173, 28.9%; χ² = 3.47, df = 2, p = 0.177, Cramér’s V = 0.08), indicating a negligible association between these variables (Table 2).

**Table 2:** Association Between Age Groups and Sex

Age group (years)	Male n (%)	Female n (%)	df	p-value	Effect size
38-58	84 (25.7%)	40 (23.1%)	2	0.177	V = 0.08
58-78	129 (39.4%)	83 (48.0%)			
≥79	114 (34.9%)	50 (28.9%)			

Regarding the overall causes of edentulism, periodontal diseases were the leading cause, affecting 234 (46.8%) of the 500 patients, followed by dental decay in 221 (44.2%) patients and other causes in 45 (9.0%) patients. When stratified by gender, males most frequently lost their teeth due to periodontal diseases (181/327, 55.4%), while dental decay was the most common cause among females (107/173, 61.8%). A Pearson Chi-square test showed that the association between gender and cause of tooth loss was statistically significant (χ² = 34.06, df = 2, p < 0.001), with a small to moderate effect size (Cramér’s V = 0.26), as summarized in Table 3.

**Table 3:** Occurrence of Overall Causes of Tooth Lost Among Participants * Statistically significant

Reason for tooth loss	Male n (%)	Female n (%)	Total prevalence (%)	df	p-value	Effect size
Periodontal diseases	181 (55.4%)	53 (30.6%)	234 (46.8%)	2	<0.001*	Cramér’s V = 0.26
Dental decay	114 (34.9%)	107 (61.8%)	221 (44.2%)			
Other causes	32 (9.8%)	13 (7.5%)	45 (9.0%)			
Total	327 (100%)	173 (100%)	500 (100%)			

The duration of edentulism among the study population varied significantly by gender (χ² = 36.35, p < 0.001; Cramér’s V = 0.27). Males were more likely to have been edentulous for 6-12 months (144/327, 44.0%) and 3-6 months (100/327, 30.6%), whereas females most commonly experienced edentulism for 3-6 months (92/173, 53.2%). Longer durations of 2-6 years were observed only in males (Table 4).

**Table 4:** Time Distribution of Being Edentulous in Individuals * Statistically significant

Period of edentulism	Male n (%)	Female n (%)	Prevalence n (%)	df	p-value	Effect size
3-6 months	100 (30.6%)	92 (53.2%)	192 (38.4%)	5	0.001*	Cramér’s V = 0.27
6-12 months	144 (44.0%)	57 (32.9%)	201 (40.2%)			
1-2 years	40 (12.2%)	18 (10.4%)	58 (11.6%)			
2-4 years	13 (4.0%)	0 (0.0%)	13 (2.6%)			
4-6 years	20 (6.1%)	0 (0.0%)	20 (4.0%)			
>10 years	10 (3.1%)	6 (3.5%)	16 (3.2%)			
Total	327 (100%)	173 (100%)	500 (100%)			

Tongue position was significantly associated with gender (χ² = 53.12, p < 0.001; Cramér’s V = 0.33), with males predominantly exhibiting Class I tongue position (265/327, 81.0%) and females more frequently showing Class III (72/173, 41.6%). In contrast, residual ridge classification showed no significant association with gender (χ² = 0.486, p = 0.784; Cramér’s V = 0.03), indicating similar distributions among males and females (Table 5).

**Table 5:** Tongue Position Prevalence and Residual Ridge Classification in Complete Edentulous Subjects * Statistically significant

Variable	Category	Male n (%)	Female n (%)	Total n (%)	χ²	df	p-value	Effect size (V)
Tongue position	Class I	265 (81.0%)	90 (52.0%)	355 (71.0%)	53.12	2	<0.001*	0.330
	Class II	1 (0.3%)	11 (6.4%)	12 (2.4%)				
	Class III	61 (18.7%)	72 (41.6%)	133 (26.6%)				
Residual ridge	Orthognathic	282 (86.2%)	153 (88.4%)	435 (87.0%)	0.486	2	0.784	0.031
	Retrognathic	29 (8.9%)	13 (7.5%)	42 (8.4%)				
	Prognathic	16 (4.9%)	7 (4.0%)	23 (4.6%)				

## Discussion

This cross-sectional observational study was conducted on 500 completely edentulous Libyan patients who required removable complete dentures. The aim was to explore associations between edentulism and patient-related factors, including age, gender, tongue position, residual alveolar ridge relationship, and duration of edentulousness.

Age is a well-established determinant in the etiology of edentulism. Elderly populations are predominantly affected and exhibit physical characteristics associated with complete tooth loss [10,11]. Edentulism is a global concern, particularly among individuals aged 65 and older, and is not restricted to developing countries [12]. Previous studies report that 11.4% of adults aged ≥50 are edentulous, with the prevalence increasing with age from 6.2% among those aged 50-59 years to 27.7% in those aged 80 and older [13]. Douglass et al. [14] noted that the prevalence of edentulism continues to rise due to aging populations and increasing numbers of older adults.

Gender also plays a significant role in edentulism. Müller et al. [15] reported higher rates of tooth loss among women, although the impact varies by country and cultural context [16-18]. Conversely, other studies found no significant gender-based differences in edentulism prevalence [19]. Social determinants, such as educational attainment, income, and access to dental care, also influence edentulism rates [20-24]. Previous study further emphasizes that gender and patient-related factors significantly affect denture satisfaction and attitudes among elderly patients [25], supporting the clinical rationale for evaluating these variables in our cohort.

In the present study, 65.4% of participants were male, and 34.6% were female. The largest age group among participants was 58-78 years (42.4%), followed by those ≥79 years (32.8%), and 38-58 years (24.8%). No statistically significant association was observed between age group and gender (χ² = 3.47, p = 0.177; Cramér’s V = 0.08), indicating a negligible relationship between these variables. These findings align with previous reports that the majority of edentulous patients fall within the older age groups [19,26,27].

Tooth loss is commonly attributed to biological disease processes such as dental caries, periodontal disease, trauma, or oral cancer [22]. While many studies identify dental caries as the primary cause, followed by periodontal disease [28], others show varying trends. For example, Hull et al. [29] reported decay as the primary cause (37%), followed by periodontal disease (29%), and trauma (12%). Alaboudi et al. [30] identified caries (89.8%) as the predominant cause, followed by trauma (4.1%) and orthodontic reasons (1.9%). Tooth loss in this cohort was primarily attributed to periodontal diseases (46.8%), followed closely by dental decay (44.2%), with other causes accounting for 9.0%. When stratified by gender, males predominantly lost teeth due to periodontal disease (55.4%), whereas females were most affected by dental decay (61.8%). The association between gender and cause of tooth loss was statistically significant (χ² = 34.06, p < 0.001; Cramér’s V = 0.26), representing a small to moderate effect size. These findings highlight the differential impact of oral diseases between genders, emphasizing the need for gender-specific preventive strategies.

Tongue position is a critical factor affecting mandibular denture stability and border seal. In edentulous individuals, the tongue may hypertrophy and occupy more space in the oral cavity [8]. Kotsiomiti and Kapari [31] reported a higher prevalence of retracted tongue position in edentulous patients compared to dentate individuals, irrespective of the duration of edentulism. Class III (retracted) tongue position complicates denture fabrication by interfering with the sublingual space and mandibular border seal [32]. Although little research has explored the relationship between tongue position and gender, tongue position is a key factor affecting mandibular denture stability and border seal. In this study, Class I tongue position was most prevalent (71.0%), particularly among males (81.0%), while Class III (retracted) posture was more common among females (41.6%). Tongue position was significantly associated with gender (χ² = 53.12, p < 0.001; Cramér’s V = 0.33), indicating a moderate effect size. These results suggest that tongue posture should be carefully considered during prosthodontic treatment planning to optimize denture stability.

The maxillomandibular relationship is essential in prosthodontics, particularly in completely edentulous patients. Previous studies stated that proper occlusal alignment contributes to denture stability and prevents excessive force on supporting tissues. In the absence of teeth, jaw relationships are recorded after occlusal rims are created [33,34]. Variations such as Class II (maxillary prognathism) or Class III (mandibular prognathism) often pose clinical challenges [35-37]. These relationships, often shaped by skeletal factors and ridge resorption, influence prosthetic outcomes [38]. The residual ridge classification was predominantly orthognathic (87.0%) in both genders, with retrognathic and prognathic relationships observed less frequently (8.4% and 4.6%, respectively). No significant association was found between residual ridge type and gender (χ² = 0.486, p = 0.784; Cramér’s V = 0.031), suggesting similar anatomical distributions among male and female patients.

The duration of edentulism is a key contributor to RRR. Some studies have claimed that a progressive bone remodeling process is most rapid during the first six months post-extraction [39-41]. Studies have shown conflicting evidence on the relationship between RRR and duration of edentulism [42-45]. Some suggest greater resorption in older individuals and women [46,47]. Fahmi [48] and Raja and Saleem [49] have also demonstrated changes in the neutral zone and molar placement based on edentulism duration. Other reports support more severe mandibular resorption in older females, even when controlling for age and duration [50,51].

While most literature does not address the correlation between gender and edentulism duration, the duration of edentulism varied significantly by gender (χ² = 36.35, p < 0.001; Cramér’s V = 0.27). Males were most frequently edentulous for 6-12 months (44.0%) and 3-6 months (30.6%), whereas females most commonly experienced edentulism for 3-6 months (53.2%). Longer durations of 2-6 years were observed exclusively in males. These findings support prior observations that duration, age, and gender influence RRR, with duration having the greatest effect [46,47,52].

This study has several notable strengths. First, the sample size was relatively large (500 patients), providing sufficient power to detect associations between gender and clinical/anatomical variables. Second, data were collected at a single center with consistent protocols, ensuring uniformity in clinical examination and data recording. Third, demographic and clinical variables, including age, gender, causes of edentulism, tongue position, and residual ridge relationships, were clearly categorized and reported. Additionally, the study accounted for regional sampling considerations and COVID-19-related delays.

Several limitations should also be acknowledged. Socioeconomic data were not collected, which may influence generalizability. Radiographic confirmation of residual ridge morphology was not performed, potentially limiting the precision of anatomical assessments. Formal inter-rater reliability metrics were not recorded; however, all clinical evaluations were performed by experienced prosthodontic faculty (assistant professors, associate professors, and professors) with joint agreement on classifications to minimize variability. The minimum three-month post-extraction enrollment criterion, while necessary to standardize soft tissue and alveolar bone healing, may restrict applicability to patients presenting earlier for prosthodontic care. Finally, the study was conducted in a public clinic providing free prosthodontic services, so findings may primarily reflect this patient population rather than all edentulous individuals in Libya. Future multi-center studies across diverse regions are warranted to validate and expand upon these findings.

## Conclusions

The present study found that complete edentulism is common among individuals with a mean age of 68 years, with males more frequently affected than females. Periodontal disease was the leading cause of tooth loss, predominantly among males, while dental decay was more prevalent in females, with small-to-moderate effect sizes. Tongue posture varied by gender, with Class I most common in males and Class III more common in females. Duration of edentulism differed by gender, with longer periods observed in males. Residual ridge relationships were predominantly orthognathic, with no significant gender differences. These findings underscore the importance of considering gender, etiology, tongue position, and duration of edentulism in prosthodontic treatment planning.
